# Risk factors of nausea and vomiting in patients with breast cancer undergoing chemotherapy: A retrospective study

**DOI:** 10.1097/MD.0000000000041067

**Published:** 2025-01-17

**Authors:** Ting Jiang, Xinming Wang, Lingli Zheng, Tao Ren, Yan Li, Jing Li

**Affiliations:** a School of Clinical Medicine, Chengdu Medical College, Chengdu, Sichuan Province, China; b Department of Pharmacy, The First Affiliated Hospital of Chengdu Medical College, Chengdu, Sichuan Province, China; c Department of Oncology, The First Affiliated Hospital of Chengdu Medical College, Chengdu, Sichuan Province, China.

**Keywords:** analysis, breast cancer, chemotherapy, nausea and vomiting, risk factors

## Abstract

Chemotherapy is one of the main treatments for breast cancer patients. However, chemotherapy-related nausea and vomiting can significantly reduce patients’ quality of life and lead to electrolyte disturbance and metabolic imbalance in severe cases. Therefore, to identify the risk factors for nausea and vomiting during chemotherapy in these patients is very important. This retrospective study included 113 patients with breast cancer undergoing chemotherapy and used antiemetic drugs hospitalized in First Affiliated Hospital of Chengdu Medical College between January 1, 2021, and January 1, 2023. Of these, 26 (23.01%) patients developed nausea and vomiting. The univariate and multivariate logistics regression analyses showed that patients combined with diabetes, hypertension, chronic hepatitis B, >65 years of age, >8 cycles of chemotherapy, and had a history of nausea and vomiting are the risk factors for nausea and vomiting. However, due to the limited sample size and some risk factor data missing from this study, further prospective studies are needed to improve and verify.

## 1. Introduction

In recent years, breast cancer was the most frequent cancer in women, and seriously affected women’s health. In 2022, 2.29 million new cases were reported, according to the latest data on the global cancer burden.^[1]^ Currently, chemotherapy is crucial in treating breast cancer, whether for early or late-stage patients. In particular, for early-stage patients, the purpose of using chemotherapy is to strive for a cure. Therefore, standardized chemotherapy is essential for patient prognosis. However, chemotherapy is often accompanied by a series of adverse reactions, among which nausea and vomiting are more common.^[2,3]^ Chemotherapy-induced nausea and vomiting (CINV) can significantly affect the quality of life of the patients and reduce their compliance with anticancer therapy, thus reducing the efficacy of the therapy.^[4]^ Therefore, the effective prevention and treatment of nausea and vomiting is a potential approach to improve anticancer treatment compliance and ensure the efficacy of chemotherapy for breast cancer patients.

However, for patients with breast cancer undergoing chemotherapy, despite the rational application of antiemetic drugs, several difficulties still exist in preventing and treating nausea and vomiting, probably owing to the incompletely clear risk factors of nausea and vomiting. Some studies have analyzed nausea and vomiting risk factors in patients with malignant tumor chemotherapy.^[5,6]^ Nevertheless, because of the extensive of the included objects and the incompleteness of the included factors, these research results lack an exact reference value for the individualized prevention and treatment of nausea and vomiting in patients with breast cancer chemotherapy.

At present, there are no systematic studies on the risk factors for nausea and vomiting in patients with breast cancer undergoing chemotherapy. Therefore, we conducted a retrospective observational study to identify its individualized risk factors for nausea and vomiting.

## 2. Materials and methods

### 2.1. Data collection

This was a retrospective observational study using data from a single center. Relevant data related to the various factors that potentially influence nausea and vomiting were collected from patients with breast cancer undergoing chemotherapy in the First Affiliated Hospital of Chengdu Medical College from January 1, 2021, to January 1, 2023. Relevant data included the patient’s age, history of alcohol consumption, number of previous chemotherapy cycles, history of nausea and vomiting, tumor stage, pathological type, molecular typing, underlying disease, concomitant pain, stage of organ dysfunction, emetic risk level of chemotherapy regimens, and method of administration of the chemotherapy regimens.

### 2.2. Inclusion and exclusion criteria

The inclusion criteria for the study were as follows: pathological diagnosis of breast cancer, application of chemotherapy, rational use of chemotherapy regimen (including rational therapeutic agent usage, rational dosage of therapeutic agents, rational schedule, and route of administration of the therapeutic agents), and rational use of antiemetic agents. The exclusion criteria for the study were as follows: key baseline data are missing, simultaneous application of radiotherapy, irrational use of chemotherapy regimen, irrational use of antiemetic drugs, pregnant women, mental illness, long-term use of glucocorticoid, long-term use of nonsteroidal anti-inflammatory drugs, and antiemetic drug usage for the treatment of other nonrelated diseases.

### 2.3. Analysis of risk factors

With the occurrence of nausea and vomiting after chemotherapy as the dependent variable and the risk factors that may affect their occurrence as the independent variable, first, single-factor logistic regression analysis was performed to screen out meaningful independent variables, followed by multivariate logistic regression analysis. In logistic regression analysis, the binary variables were assigned 0 and 1. For unordered multiclass variables, dummy variables were treated first and then assigned to consecutive numbers, such as 0, 1, 2, 3, and so on.

### 2.4. Statistical methods

All data were statistically analyzed using SPSS 23.0 software. To prevent some meaningful independent variables from being excluded in the univariate analysis, the test level was set as *P* <.10, and as long as it was <.10, it was considered significant, and then multivariate analysis was conducted. In multivariate analysis, *P* <.05 was considered statistically significant. In addition, before performing multivariate logistic regression analysis, the variance inflation factor was calculated to determine whether there is multicollinearity between explanatory variables. If multicollinearity (variance inflation factor ≥ 10) was observed, multivariate logistic regression analysis was performed using only 1 explanatory variable.

## 3. Results

### 3.1. Patient characteristics

Data from 314 patients were collected in this research, and 113 patients were finally included after screening and exclusion. The details of the excluded cases were as follows: patients with missing key baseline data (47 cases), patients who received radiotherapy simultaneously (83 cases), patients who used glucocorticoids for a prolonged period (26 cases), and patients who used antiemetic drugs for the treatment of other unrelated diseases (45 cases). Among the 113 cases included, the average age was 51.66 ± 9.91 years, and the previous cycles of chemotherapy were 6.08 ± 3.59 cycles. A total of 0.88%, 16.81%, 10.62%, 4.42%, 1.77%, and 23.01% of patients reported drinking history, underlying disease, history of nausea and vomiting, concomitant pain, organ dysfunction, and suffering from nausea and vomiting after chemotherapy. The characteristics of the patients are provided in Table 1. Critical baseline data are missing.

**Table 1:** Characteristics summary of included cases.

Objective of chemotherapy	Cases (%)	Pathological type	Cases (%)
Neoadjuvant chemotherapy	5 (4.42)	Ductal carcinoma	95 (84.07)
Adjuvant chemotherapy	99 (87.61)	Lobular carcinoma	11 (9.73)
Palliative chemotherapy	9 (7.97)	Mucous carcinoma	3 (2.65)
Patient age (years)	51.66 ± 9.91	Mixed carcinoma	3 (2.65)
Drinking history		Mastoid carcinoma	1 (0.88)
Yes	1 (0.88)	Underlying disease	
No	112 (99.12)	Yes	19 (16.81)
Number of previous cycles of chemotherapy	6.08 ± 3.59	No	94 (83.19)
History of nausea and vomiting		Concomitant pain	
Yes	12 (10.62)	Yes	5 (4.42)
No	101 (89.38)	No	108 (95.58)
Neoplasm staging		Organ dysfunction	
Phase IA	25 (22.12)	Yes	2 (1.77)
Phase IIA	29 (25.66)	No	111 (98.23)
Phase IIB	20 (17.70)	Emetic risk level of chemotherapy regimens	
Phase IIIA	15 (13.27)	High	15 (13.27)
Phase IIIB	7 (6.19)	Moderate	94 (83.19)
Phase IIIC	7 (6.19)	Low	4 (3.54)
Phase IV	10 (8.85)	Method of chemotherapy regimens administration	
Molecular typing		Once	101 (89.38)
ER(+), PR(+), HER2(3+)	18 (15.93)	Divided into 2 days	11 (9.73)
ER(−), PR(−), HER2(3+)	14 (12.39)	Divided into 3 days	1 (0.88)
ER(−), PR(−), HER2(−)	29 (25.66)	Occurrence of nausea and vomiting after chemotherapy	
ER(+), PR(+), HER2(−)	40 (35.40)	Yes	26 (23.01)
ER(+), PR(−), HER2(−)	6 (5.31)	No	87 (76.99)
ER(+), PR(−), HER2(3+)	4 (3.54)		
ER(−), PR(+), HER2(−)	2 (1.77)		

### 3.2. Univariate and multivariate logistic regression analysis results

The results of univariate logistics regression analysis revealed that the patient’s age, number of previous chemotherapy cycles, history of nausea and vomiting, and underlying diseases were the statistically significant factors for nausea and vomiting in patients with breast cancer undergoing chemotherapy. These statistically significant factors were then subjected to multivariate logistics regression analysis, and the results reveal that the patient’s age (odds ratio [OR]: 4.541, 95% confidence interval [CI]: 1.142–8.051, *P* = .032), number of previous chemotherapy cycles (OR: 8.307, 95% CI: 2.128–32.435, *P* = .002), history of nausea and vomiting (OR: 18.043, 95% CI: 4.176–77.956, *P* = .000) and underlying disease (OR: 3.702, 95% CI: 1.074–12.768, *P* = .038) were statistically significant risk factors for nausea and vomiting in patients with breast cancer undergoing chemotherapy. The results of the analysis are shown in Table 2.

**Table 2:** Logistics regression analysis of CINV occurrence in patients with breast cancer undergoing chemotherapy.

Predictor	Cases	CINV occurred	Univariate analysis		Multivariate analysis	
		Cases (%)	OR (95% Cl)	*P* value	OR (95% Cl)	*P* value
Objective of chemotherapy						
Neoadjuvant chemotherapy	5	0 (0.00)				
Adjuvant chemotherapy	99	21 (21.21)	0.000	.999		
Palliative chemotherapy	9	5 (55.56)	0.357 (0.088–1.445)	.149		
Patient age			4.211 (1.319–13.444)	.015*	4.541 (1.142–18.051)	.032*
≤65	99	19 (19.19)				
>65	14	7 (50.00)				
Drinking history			0.000	1.000		
Yes	1	0 (0.00)				
No	112	26 (23.21)				
Number of previous cycles of chemotherapy			4.974 (1.499–16.504)	.009*	8.307 (2.128–32.435)	.002*
≤8	100	19 (19.00)				
>8	13	7 (53.85)				
History of nausea and vomiting			9.222 (2.503–33.972)	.001*	18.043 (4.176–77.956)	.000*
Yes	12	8 (66.67)				
No	101	18 (17.82)				
Neoplasm staging						
Phase IA	25	4 (16.00)				
Phase IIA	29	8 (27.59)	2.000 (0.522–7.669)	.312		
Phase IIB	20	3 (15.00)	0.926 (0.182–4.718)	.927		
Phase IIIA	15	3 (20.00)	1.312 (0.250–6.879)	.748		
Phase IIIB	7	2 (28.57)	2.100 (0.297–14.873)	.458		
Phase IIIC	7	2 (28.57)	2.100 (0.297–14.873)	.458		
Phase IV	10	4 (40.00)	3.500 (0.668–18.343)	.138		
Pathological type						
Ductal carcinoma	95	23 (24.21)				
Lobular carcinoma	11	2 (18.19)	0.313 (0.038–2.578)	.280		
Mucous carcinoma	3	1 (33.33)	1.565 (0.136–18.065)	.720		
Mixed carcinoma	3	0 (0.00)	1.565 (0.136–18.065)	.720		
Mastoid carcinoma	1	0 (0.00)	0.000	1.000		
Molecular typing						
ER(+), R(+), HER2(3+)	18	3 (16.67)				
ER(−), PR(−), HER2(3+)	14	4 (28.57)	2.000 (0.366–10.919)	.423		
ER(−), PR(−), HER2(−)	29	8 (27.59)	1.905 (0.432–8.394)	.394		
ER(+), PR(+), HER2(−)	40	10 (25.00)	1.667 (0.398–6.974)	.484		
ER(+), PR(−), HER2(−)	6	1 (16.67)	1.000 (0.084–11.931)	1.000		
ER(+), PR(−), HER2(3+)	4	0 (0.00)	0.000	.999		
ER(−), PR(+), HER2(−)	2	0 (0.00)	0.000	.999		
Underlying disease			5.417 (1.897–15.463)	.002*	3.702 (1.074–12.768)	.038*
Yes	19	10 (52.63)				
No	94	16 (17.02)				
Concomitant pain			0.000	.999		
Yes	5	0 (0.00)				
No	108	26 (24.07)				
Organ dysfunction			3.440 (0.208–56.987)	.388		
Yes	2	1 (50.00)				
No	111	25 (22.52)				
Emetic risk level of chemotherapy regimens						
High	15	5 (33.33)				
Moderate	94	19 (20.21)	0.507 (0.155–1.658)	.261		
Low	4	2 (50.00)	2.000 (0.214–18.687)	.543		
Method of chemotherapy regimen administration						
Once	101	24 (23.76)				
Divided into 2 days	11	2 (18.18)	0.713 (0.144–3.529)	.678		
Divided into 3 days	1	0 (0.00)	0.000	1.000		

CI, confidence interval, CINV = chemotherapy-induced nausea and vomiting, OR = odds ratio.

* Statistically significant.

## 4. Discussion

### 4.1. Risk factors for nausea and vomiting

The occurrence of nausea and vomiting in chemotherapy patients is associated with multiple risk factors, such as a history of nausea and vomiting.^[7]^ Some studies have indicated that the age of patients is related to the occurrence of nausea and vomiting. Kawazoe et al^[8]^, Simino et al^[9]^, and Warr^[10]^ reported that younger patients are more likely to occur CINV than older patients. However, some studies have shown older patients have an increased risk of acute side effects such as nausea and vomiting.^[11]^

### 4.2. Key findings of our study

The average age of the patients included in this research was 51.66 ± 9.91 years. The results showed that more than 65 years of age was a risk factor for nausea and vomiting, which was consistent with the results of the study by Hesketh et al.^[12]^ The possible reason was that the use of antiemesis agents changed the risk of CINV associated with age. Some studies have suggested that drinking history can reduce the incidence of nausea and vomiting in patients with CINV.^[12,13]^ However, because the subjects included in this study were all female, the number of cases with a drinking history is minimal. Therefore, the univariate analysis results cannot indicate that drinking is a risk factor for the occurrence of nausea and vomiting. Furthermore, recent studies have shown that patients with a history of nausea and vomiting are more prone to nausea and vomiting, possibly because previous episodes of nausea and vomiting render the related reflex pathway more active; therefore, they are more likely to suffer nausea and vomiting again.^[14]^ The results of our study are consistent with this, and the risk of recurrence in patients with a history of nausea and vomiting is 9.222 times higher than that in patients without a history of nausea and vomiting. Some studies have shown that the incidence of CINV (delayed and acute) decreases with the increased number of previous chemotherapy cycles,^[15]^ while some studies have shown that the incidence of anticipated vomiting will increase with the increased number of previous chemotherapy cycles.^[16]^ The average number of chemotherapy cycles included in this research was 6.08 ± 3.59 cycles. The results showed that more than 8 cycles of chemotherapy are also a risk factor for nausea and vomiting. Herein, the *P*-values of other factors, such as tumor stage, and molecular and pathological types, were all >0.1. Therefore, they did not have a statistically significant difference in the occurrence of nausea and vomiting. In addition, concomitant diseases or organ dysfunction also may induce or aggravate the occurrence of nausea and vomiting in patients. Some studies have shown that the incidence of nausea and vomiting in patients with pain can be induced or aggravated,^[17,18]^ and some studies have also demonstrated that nausea is more common in patients with liver metastasis.^[19]^ However, no effective results could be obtained due to the limited number of patients with pain and liver metastasis included in this research. But, the results of the current study showed that patients with diabetes, hypertension, and chronic hepatitis B had a higher risk of nausea and vomiting, which was 5.417 times higher than patients without these diseases.

### 4.3. Novelty and limitation of our study

In this study, the risk factors of nausea and vomiting in patients with breast cancer chemotherapy were comprehensively analyzed. The obtained results can provide a more accurate reference for preventing and treating nausea and vomiting in these patients during chemotherapy. However, the current research also has some limitations, primarily related to 3 aspects: First, the sample size of included patients is limited, which may influence the robustness of the results; Second, for missing data, we used the method of direct deletion, which may lead to the loss of some valuable information and reduce the accuracy of the results; Third, the data related to some risk factors in the case data are missing, which may lead to incomplete analysis. For example, current studies have shown that morning sickness, motion sickness, and anxiety are all risk factors for nausea and vomiting.^[20]^ The risk of CINV is even related to the polymorphisms of 5-HT3 receptor and metabolizers of 5-HT3 receptor antagonists.^[21,22]^

### 4.4. Future research directions

Given the limitations of this study, the follow-up study will expand the sample size, include the analysis of more influencing factors, and conduct prospective studies, to obtain more reliable research results.

## Acknowledgments

We thank Medicine for providing a platform for sharing clinical research results.

## Author contributions

**Conceptualization:** Ting Jiang, Tao Ren, Jing Li, Xinming Wang, Lingli Zheng.

**Investigation:** Ting Jiang, Lingli Zheng.

**Methodology:** Ting Jiang, Yan Li.

**Software:** Ting Jiang.

**Writing – original draft:** Ting Jiang.

**Validation:** Tao Ren, Yan Li.

**Data curation:** Jing Li.

**Formal analysis:** Jing Li, Xinming Wang.

**Project administration:** Jing Li, Yan Li.

**Resources:** Jing Li, Lingli Zheng.

**Visualization:** Jing Li.

**Writing – review & editing:** Jing Li.
